# Pre-operative anxiety in the ophthalmological surgery

**DOI:** 10.1192/j.eurpsy.2023.229

**Published:** 2023-07-19

**Authors:** S. V. Kuzmina, A. Y. Rascheskov, N. D. Kuzmin

**Affiliations:** 1 Psychiatry and medical psychology department, KAZAN STATE MEDICAL UNIVERSITY; 2 Head of ophthalmology clinic, Eye surgery Rascheskov; 3 Resident of Ophthalmology, The S. Fyodorov Eye Microsurgery Federal State Institution of the Ministry of Health of Russia, Kazan, Russian Federation

## Abstract

**Introduction:**

The question of preoperative anxiety in an ophthalmological patient and its relief in an outpatient setting is becoming increasingly relevant due to the improvement of the technique and equipment of ophthalmic surgery, when the practice of «one-day surgery» is becoming increasingly common. It could not only affecting the course of the operation, but also a factor limited the availability of this type of medical care to patients. **Purpose.** To analyze the relationship between the level of anxiety during preoperative preparation and the choice of the optimal approach for the relief of preoperative anxiety in an ophthalmological patient in a «one day» clinic.

**Objectives:**

89 adult patients of both sexes aged from 20 y.o. referred for outpatient surgery (refractive and cataract surgery) and were included into trials, all of them are divided into main - 45 and control - 44 groups. Clinical psychotherapeutic interviewing, a scale of situational and personal anxiety C.D. Spielberger (adapted by Y.L. Khanin), a questionnaire for analyzing satisfaction with the quality of medical services provided in outpatient settings were used. Statistical: Microsoft Excel spreadsheet editor for Windows; the STATISTICA application software package version 6.1. were taken. The condition for determining statistically significant differences is the value р≤0,05.

**Methods:**

Two-stage study by the method of contiuous sampling. Clinical psychotherapeutic interviewing, a scale of situational and personal anxiety C.D. Spielberger (adapted by Y.L. Khanin), a questionnaire for analyzing satisfaction with the quality of medical services provided in outpatient settings were used. Statistical: Microsoft Excel spreadsheet editor for Windows; the STATISTICA application software package version 6.1. were taken. The condition for determining statistically significant differences is the value р≤0,05.

**Results:**

The level of anxiety in patients who received anxiolytics

**Table:** 

Level of anxiety	examination day, %	day of operation %		
	SA	PA	SA	PA
low	22*	22,5	**35** *	21
medium	53,5	64,5	57	67
high	24,5*	13	**8** *	13

* - р≤0,05; SA-situational anxiety; PS – personal anxiety

The study revealed an average and high level of situational anxiety in 66% of patients referred for refractive surgery, in 81% of patients referred for cataract surgery. Into main group, against the background of the performed anxiolytic therapy in the preoperative period, the proportion of people with a high level of reactive anxiety decreased significantly (p≤0.05) (from 24% to 8%), while personal anxiety did not significantly change. Among control group patients show a lower level of satisfaction with the quality of medical services provided in an outpatient setting.

**Conclusions:**

The study showed the possibility of providing better quality medical services in «one-day» eye surgery, which expands the availability of outpatient ophthalmic surgical care to patients with high level of anxiety and anxiety disorders.

**Disclosure of Interest:**

None Declared

